# Supplementary material for: Netcombin: An algorithm for constructing optimal phylogenetic network from rooted triplets
Source: PLoS One. 2020 Sep 18;15(9):e0227842. doi: 10.1371/journal.pone.0227842 (PMC7500971; doi:10.1371/journal.pone.0227842)
Supplement: S4 File — (ZIP) [file pone.0227842.s004.zip › SequenceData-Triplet/sequenceData-10-20-30-0-Triplet/length 200, numseq 30, rate 10^-7/3 seq.pdf]

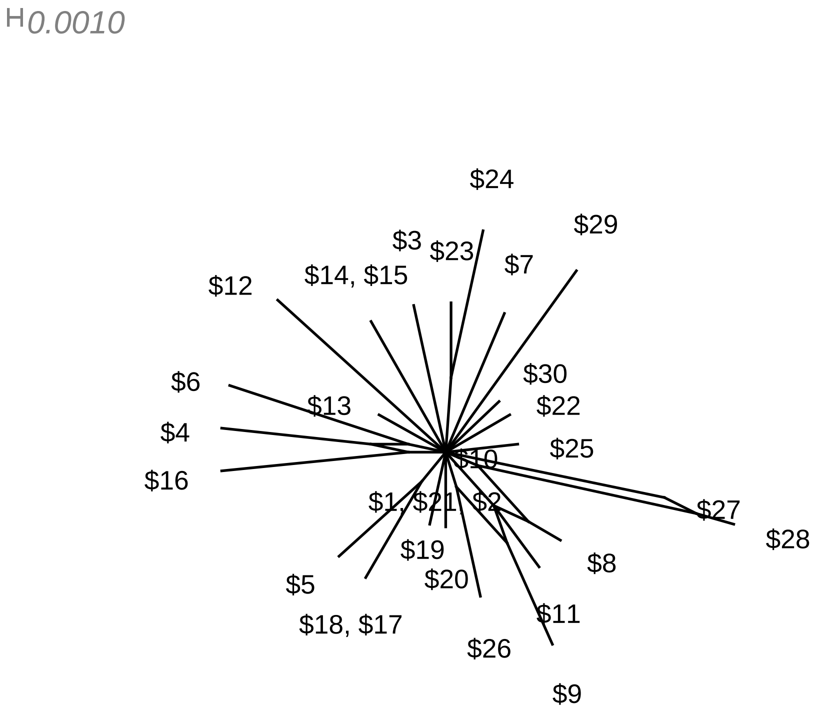

H0.0010
$24
$29
$3
$23
$7
$14, $15
$12
$30
$6
$13
$22
$4
$25
$10
$16
$27
$1, $21, $2
$28
$19
$8
$5
$20
$18, $17
$11
$26
$9
